# Temporal Progression Patterns of Brain Atrophy in Corticobasal Syndrome and Progressive Supranuclear Palsy Revealed by Subtype and Stage Inference (SuStaIn)

**DOI:** 10.3389/fneur.2022.814768

**Published:** 2022-02-25

**Authors:** Yuya Saito, Koji Kamagata, Peter A. Wijeratne, Christina Andica, Wataru Uchida, Kaito Takabayashi, Shohei Fujita, Toshiaki Akashi, Akihiko Wada, Keigo Shimoji, Masaaki Hori, Yoshitaka Masutani, Daniel C. Alexander, Shigeki Aoki

**Affiliations:** ^1^Department of Radiology, Juntendo University Graduate School of Medicine, Tokyo, Japan; ^2^Centre for Medical Image Computing, Department of Computer Science, University College London, London, United Kingdom; ^3^Department of Radiology, Graduate School of Medicine, University of Tokyo, Tokyo, Japan; ^4^Department of Radiology, Tokyo Metropolitan Geriatric Hospital and Institute of Gerontology, Tokyo, Japan; ^5^Department of Radiology, Toho University Omori Medical Center, Tokyo, Japan; ^6^Department of Biomedical Information Sciences, Hiroshima City University Graduate School of Information Sciences, Hiroshima, Japan

**Keywords:** brain atrophy, disease progression, machine learning, corticobasal syndrome, corticobasal degeneration, progressive supranuclear palsy, magnetic resonance imaging, classification

## Abstract

Differentiating corticobasal degeneration presenting with corticobasal syndrome (CBD-CBS) from progressive supranuclear palsy with Richardson's syndrome (PSP-RS), particularly in early stages, is often challenging because the neurodegenerative conditions closely overlap in terms of clinical presentation and pathology. Although volumetry using brain magnetic resonance imaging (MRI) has been studied in patients with CBS and PSP-RS, studies assessing the progression of brain atrophy are limited. Therefore, we aimed to reveal the difference in the temporal progression patterns of brain atrophy between patients with CBS and those with PSP-RS purely based on cross-sectional data using Subtype and Stage Inference (SuStaIn)—a novel, unsupervised machine learning technique that integrates clustering and disease progression modeling. We applied SuStaIn to the cross-sectional regional brain volumes of 25 patients with CBS, 39 patients with typical PSP-RS, and 50 healthy controls to estimate the two disease subtypes and trajectories of CBS and PSP-RS, which have distinct atrophy patterns. The progression model and classification accuracy of CBS and PSP-RS were compared with those of previous studies to evaluate the performance of SuStaIn. SuStaIn identified distinct temporal progression patterns of brain atrophy for CBS and PSP-RS, which were largely consistent with previous evidence, with high reproducibility (99.7%) under cross-validation. We classified these diseases with high accuracy (0.875) and sensitivity (0.680 and 1.000, respectively) based on cross-sectional structural brain MRI data; the accuracy was higher than that reported in previous studies. Moreover, SuStaIn stage correctly reflected disease severity without the label of disease stage, such as disease duration. Furthermore, SuStaIn also showed the genialized performance of differentiation and reflection for CBS and PSP-RS. Thus, SuStaIn has potential for improving our understanding of disease mechanisms, accurately stratifying patients, and providing prognoses for patients with CBS and PSP-RS.

## Introduction

Corticobasal degeneration (CBD) and progressive supranuclear palsy (PSP) are sporadic atypical parkinsonian disorders associated with the accumulation of insoluble deposits of predominantly four microtubule-binding domain repeat (4R) tau protein in specific central nervous system neurons and glia (1–4). CBD has a variety of phenotypes, and pathological symptoms depend on tau lesions in the frontoparietal cortex, particularly in the primary motor and somatosensory cortices (5, 6). The most common clinical syndrome of CBD is corticobasal syndrome (CBS), which is characterized by Parkinsonism, rigidity, unilateral dystonia, myoclonus, alien limb, and ideomotor apraxia (7). In contrast, PSP is a classical Richardson's syndrome with symptoms of postural instability and vertical supranuclear gaze palsy (8); it is pathologically characterized by tau lesions mainly in the midbrain and superior cerebellar peduncle (SCP), especially in the substantia nigra and dentate nucleus (9). Currently, effective treatments for CBS or PSP-RS are not available. Moreover, the etiology and onset mechanism remain poorly understood. Furthermore, the neurodegenerative conditions closely overlap in terms of clinical information, pathology, biochemistry, and genetic risk factors; thus, differentiating CBD presenting with CBS (CBD-CBS) from PSP-RS, particularly in early stages, is often difficult (10). Several morphological markers on MRI, including the “hummingbird” sign of midbrain atrophy compared with pons and the “morning glory” sign of midbrain tegmentum atrophy (11–13), can indicate PSP-RS. However, in the study of MR findings before autopsy confirmation, these signs have high specificity but low sensitivity [“hummingbird” sign (sensitivity: ~51.0%; specificity: ~99.5%] and “morning glory” sign (sensitivity: ~37.0%; specificity: ~97.0%)] for distinguishing PSP-RS from CBD-CBS (14). However, accurate differentiation between PSP-RS and CBS is important to facilitate the early diagnosis of PSP-RS and CBS for the accurate prognostication and stratification of patients for clinical trials.

Numerous studies have evaluated MRI-based brain volumetry data to distinguish CBS from PSP-RS based on the specific patterns of brain atrophy. Brain atrophy in CBS primarily involves the frontoparietal lobe, especially the pre- and postcentral gyri (15–21). In contrast, in patients with PSP-RS, brain atrophy is most prominent in the brainstem regions, particularly in the midbrain tegmentum and SCP (15–19, 22, 23). Although revealing the temporal progression patterns of brain atrophy may help understand disease mechanisms and enable more accurate patient stratification and prognostication, most studies evaluating brain MRI data in patients with CBS and PSP-RS were cross-sectional in nature. A significant amount of longitudinal data is required to evaluate disease progression, and the collection of such data adds a considerable burden in terms of time, effort, and money. Moreover, tracking large populations is challenging. Although a few studies have investigated longitudinal brain atrophy using longitudinal brain structural MRI at some time points (e.g., baseline, over 6 months and 1 year) based on the assumption that volumetric changes are linear (24–26), they failed to identify temporal atrophy progression with over a few disease stages or provide more information on the longitudinal atrophy of patients with CBS and PSP-RS.

The accuracy of MRI-based brain volumetry has not always been higher than that of clinical criteria; sample sizes were often small, and its use for single-subject decision-making was limited. Therefore, various classifiers based on MRI-based brain volumetry have been proposed for differentiating patients with PSP-RS from those with CBS. In a previous study, Correia et al. used a support vector machine (SVM)—a statistical classifier—and gray matter volume data to classify 19 patients with PSP-RS and 19 patients with CBS; however, the classification accuracy was only 62.2% (27). Gröschel et al. (16) used a mathematical model for brain MR volumetry, including the midbrain, parietal white matter, temporal gray matter, brainstem, frontal white matter, and pons, in patients with PSP-RS (*n* = 33) and CBS (*n* = 18) and achieved a classification accuracy of 79.5%. Therefore, it is challenging to precisely differentiate between CBS and PSP-RS even using the latest methods. The low accuracy of differentiation between patients with CBS and those with PSP-RS might have resulted from developing the model with an aim to only predict disease subtypes, without considering the disease-stage heterogeneity (28).

**Figure 1:**
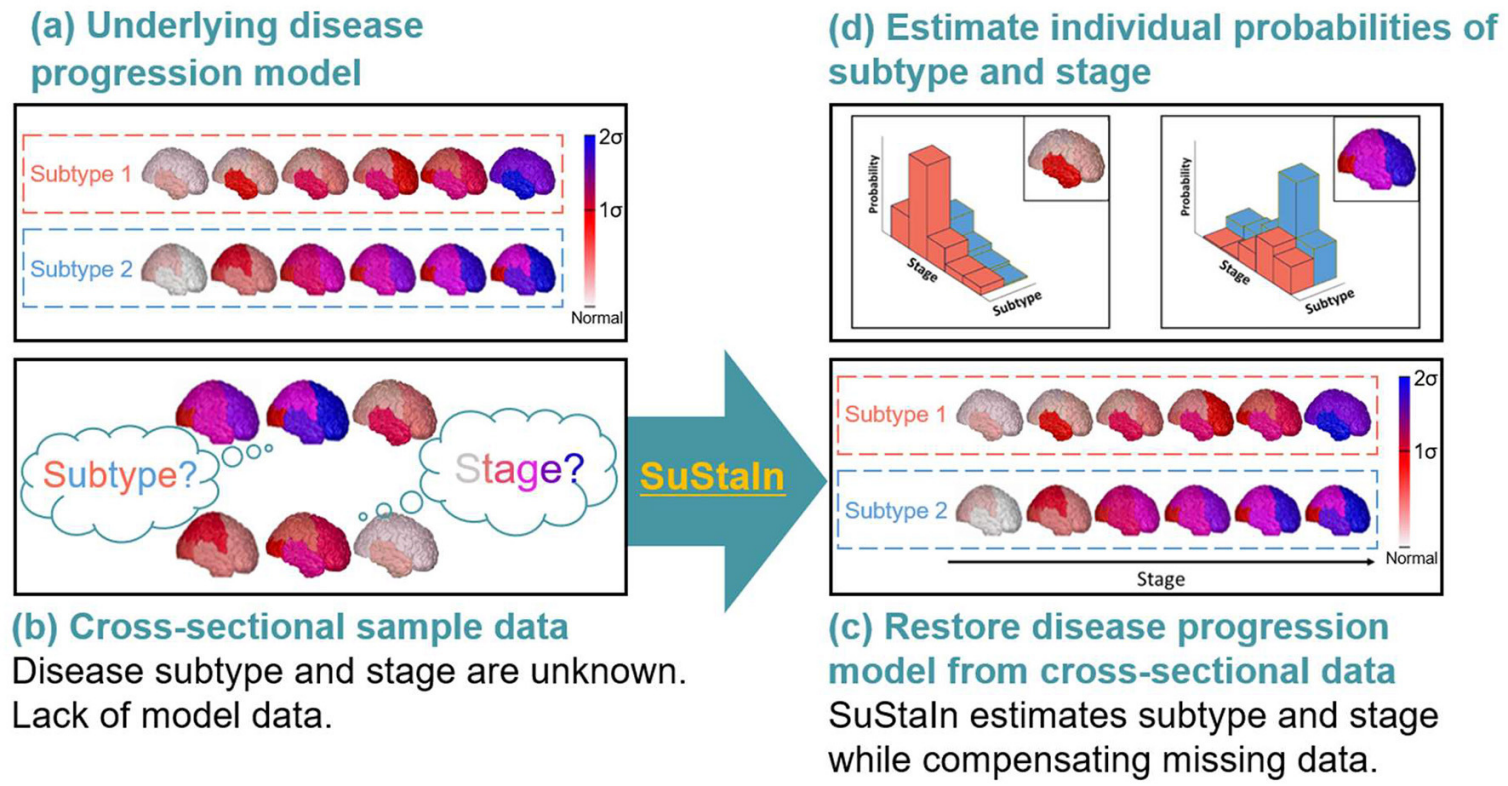
Conceptual overview of SuStaIn modeling. Assuming the underlying model **(a)**, cross-sectional sample data contained biomarker measurements from each subject with an unknown disease subtype and stage **(b)**. SuStaIn restored disease subtypes and temporal progression *via* simultaneous clustering and disease progression modeling **(c)**. Moreover, SuStaIn estimated the probability that a subject belonged to each subtype and stage based on a reconstructed snapshot **(d)**. The color of each region indicates the severity of pathology, which ranges from white to red, to magenta, to blue.

Despite the development of disease progression and classifier models for CBS and PSP-RS using MRI-based brain volumetric data, to our knowledge, no model has been developed that can integrates and simultaneously estimates disease progression as well as can differentiate CBS and PSP-RS based on the longitudinal and low amount of cross-sectional brain structural MRI regional brain volume data. Recently, an unsupervised machine learning technique called Subtype and Stage Inference (SuStaIn) (28) was developed to identify data-driven disease phenotypes with distinct temporal progression patterns (Figure 1). The technique integrates clustering and disease progression modeling based on widely available cross-sectional data (Figures 1a–c). Thus, compared with models that only predict disease subtypes, SuStaIn can model disease progression using only cross-sectional, but not longitudinal, data and disease-stage heterogeneity to allow better stratification of patients with CBS and those with PSP-RS (28). If the sample size of the input cross-sectional data (Figure 1b) is insufficient for reconstructing the underlying disease progression model (Figure 1a), SuStaIn can restore disease progression model from the insufficient input data (Figure 1c) and estimate individual probabilities of disease subtypes and stages (Figure 1d). Young et al. (28) applied SuStaIn to reveal disease subtypes and distinct trajectories of regional brain atrophy-based MR volumetry in genetic frontotemporal dementia (FTD) and Alzheimer's disease (AD). As a validation, SuStaIn correctly identifies distinct genetic subtypes of FTD without seeing the genetic information. In sporadic AD, the algorithm identified three subtypes that correspond to end-stage patterns observed in postmortem pathology. Moreover, SuStaIn provided good separation between presymptomatic and symptomatic mutation carriers of FTD, cognitively normal patients, and patients with AD. Thus, SuStaIn may enable the simultaneous identification of disease subtypes and allow inferences on the progression of each subtype.

In this study, we applied SuStaIn to cross-sectional brain structural MRI regional brain volume data to identify differences in the temporal progression patterns of brain atrophy between patients with CBS and PSP-RS. We hypothesized that SuStaIn is useful in assessing and differentiating the progressions of CBS and PSP-RS.

## Methods

### Study Cohorts

The data used in the preparation of this manuscript were obtained from the 4-Repeat Tauopathy Neuroimaging Initiative (4RTNI) and the Frontotemporal Lobar Degeneration Neuroimaging Initiative (FTLDNI) database. The primary goal of the 4RTNI is to identify neuroimaging and biomarker indicators for disease progression in 4R tauopathy neurodegenerative diseases, PSP-RS, and CBD. The primary goals of FTLDNI are to identify neuroimaging modalities and analysis methods for tracking frontotemporal lobar degeneration and assess the value of imaging vs. other biomarkers for diagnosis. Both initiatives are managed by the University of California San Francisco (UCSF) and follow the same principal study design and protocol. Detailed information on 4RTNI and FTLDNI is available at http://4rtni-ftldni.ini.usc.edu/. In this study, we used two datasets of different sites and MR acquisition parameters as (internal dataset: 114 participants, external dataset: 17 participants).

As internal dataset, data of 25 patients with CBS and 39 with PSP-RS were obtained from the 4RTNI database. Data of 50 healthy controls (HCs) were obtained from the FTLDNI database. This study included the data of age-, sex-, and disease duration-matched participants recruited at the UCSF during their first visit and who were scanned using the same MRI acquisition parameters (see Methods: MRI acquisition) as those used for participants to adjust for any effects due to the differences in MRI acquisition site and parameters (29). To evaluate generalization performance of SuStaIn, as external dataset, data of 5 patients with CBS and 12 with PSP-RS, who were recruited at 3 sites [University of Toronto (Toronto); University of California, San Diego (UCSD); Massachusetts General Hospital [MGH]] during their first visit and who were scanned using the same MRI acquisition parameters, were obtained from the 4RTNI database.

All participants underwent a comprehensive neurological examination, which included the following: the PSP Rating Scale (PSPRS) ranging from 0 (best) to 100 (worst) (30), four motor subscores of total PSPRS, including PSPRS Bulbar, PSPRS Ocular Motor, PSPRS Limb Motor, and PSPRS Gait/Midline (30) and Unified Parkinson's Disease Rating Scale (UPDRS) ranging from 0 (best) to 108 (worst) (31) for evaluating motor function; the Mini-Mental State Examination (MMSE) ranging from 0 (worst) to 30 (best) (32) and Montreal Cognitive Assessment (MoCA) ranging from 0 (worst) to 30 (best) (33) for evaluating cognitive function; the Clinical Dementia Rating (CDR) Box ranging from 0 (best) to 18 (worst) (34), the Schwab and England Activities of Daily Living (SEADL) ranging from 0% (worst) to 100% (best) (35), and the Functional Activities Questionnaire (FAQ) ranging from 0 (best) to 30 (worst) (36) for evaluating abilities of daily living activities. Patients with PSP-RS met the National Institute of Neurological Disorders and Stroke for PSP (NINDS-SPSP) criteria for PSP-Richardson's syndrome (AL-108-231) (8, 37). Patients with CBS met the Armstrong's criteria for possible or probable CBD-CBS subtype (7). Patients with CBS and PSP-RS who had no motor symptoms were excluded. All HCs were cognitively normal with an MMSE score of ≥27 and a MoCA score of ≥25 and had no impairments in the activities of daily living with a CDR Box score of ≤1, FAQ score of ≤1, and a SEADL score of 100%. Autopsy-confirmed diagnosis was available for two patients with CBD-CBS and two patients with PSP-RS. Furthermore, T1-weighted images of all participants were evaluated by a neuroradiologist (K.K.) with >10 years of experience in MRI of neurodegenerative disease to assess the classical MR findings of the “asymmetrical frontoparietal atrophy” in CBS (15–21) and “hummingbird” sign in PSP-RS (11–13). Population demographics and clinical characteristics are shown in Table 1.

**Table 1:** Demographic characteristics of participants.

**Information**	**HC**	**CBS**	**PSP-RS**	**HC vs. CBS/PSP-RS *P*-value**	**CBS vs. PSP-RS *P*-value**
**(a) Internal dataset**
No. of MRI scans	50	25	39	—	—
Age, y	68.7 ± 4.0	67.8 ± 5.2	68.8 ± 7.1	*n.s*.	*n.s*.
Sex, % male	52.0	52.0	53.8	*n.s*.	*n.s*.
Disease duration, y	—	5.3 ± 2.8	4.8 ± 3.1	—	*n.s*.
Autopsy-confirmed subjects	—	2	2	—	—
PSPRS total	—	25.6 ± 9.5	36.0 ± 16.0	—	<0.05^*^
PSPRS bulbar	—	1.1 ± 1.0	2.6 ± 1.5	—	<0.001^****^
PSPRS ocular motor	—	1.9 ± 1.9	7.4 ± 3.6	—	<0.001^****^
PSPRS limb motor	—	8.7 ± 3.2	4.8 ± 2.5	—	<0.001^****^
PSPRS gait/midline	—	6.0 ± 4.9	9.8 ± 5.3	—	<0.05^*^
UPDRS-III total	—	35.4 ± 16.0	30.8 ± 16.5	—	*n.s*.
CDR box	0.1 ± 0.2	3.4 ± 2.9	3.7 ± 3.0	<0.001^****^	*n.s*.
MMSE total	29.4 ± 0.8	23.8 ± 5.5	25.2 ± 3.6	<0.001^****^	*n.s*.
MoCA total	27.4 ± 1.3	19.7 ± 6.5	21.3 ± 3.8	<0.001^****^	*n.s*.
FAQ total	0.0 ± 0.2	11.2 ± 7.0	14.1 ± 7.7	<0.001^****^	*n.s*.
SEADL, %	100.0 ± 0.0	56.8 ± 16.9	55.8 ± 27.2	<0.001^****^	*n.s*.
**MRI findings**
Asymmetrical frontoparietal atrophy, % (*n*)	0% (0/50)	84.0% (21/25)	23.1% (9/39)	—	—
Hummingbird sign, % (*n*)	0% (0/50)	16.0% (4/25)	64.1% (25/39)	—	—
**Information**		**CBS**	**PSP-RS**		**CBS vs. PSP-RS** ***P*****-value**
**(b) External dataset**
No. of MRI scans		5	12		—
Age, y		68.8 ± 9.2	71.0 ± 7.5		*n.s*.
Sex, % male		60.0	50.0		*n.s*.
Disease duration, y		*none*	*none*		—
Autopsy confirmed subjects		0	0		—
PSPRS Total		24.6 ± 6.6	37.2 ± 19.1		*n.s*.
PSPRS Bulbar		2.2 ± 1.2	2.4 ± 1.3		*n.s*.
PSPRS Ocular–Motor		1.8 ± 1.5	7.3 ± 4.0		<0.01^**^
PSPRS Limb–Motor		7.8 ± 2.9	4.9 ± 3.3		*n.s*.
PSPRS Gait/Midline		4.0 ± 10.5	10.5 ± 6.1		*n.s*.
UPDRS-III Total		32.8 ± 7.4	26.8 ± 13.0		*n.s*.
CDR Box		4.8 ± 4.5	5.8 ± 4.5		*n.s*.
MMSE		18.8 ± 10.6	18.8 ± 7.0		*n.s*.
MoCA Total		21.3 ± 11.6	24.5 ± 7.9		*n.s*.
FAQ		11.8 ± 9.5	12.0 ± 10.0		*n.s*.
SEADL, %		52.0 ± 19.4	60.9 ± 32.9		*n.s*.
**MRI findings**
Asymmetrical frontoparietal atrophy, % (*n*)		80.0% (4/5)	27.3% (3/12)		—
Hummingbird sign, % (*n*)		20.0% (1/5)	63.6% (8/12)		—

*HC, healthy control; CBS, corticobasal syndrome; PSP, progressive supranuclear palsy; PSPRS, PSP Rating Scale; Four motor subscores from total PSPRS, PSPRS Bulbar, PSPRS Ocular–Motor, PSPRS Limb–Motor, and PSPRS Gait/Midline; UPDRS-III, Part-III (motor exams) of the Unified Parkinson's Disease Rating Scale; CDR Box, Clinical Dementia Rating Sum of Boxes; MMSE, Mini-Mental State Examination; MoCA, Montreal Cognitive Assessment; FAQ, Functional Activities Questionnaire; SEADL, Schwab and England Activities of Daily Living; n.s., not significant*.

* *: P < 0.05*,

** *: P < 0.01*,

^***^ : P < 0.005,

**** *: P < 0.001*.

### MRI Acquisition

For internal dataset, three-dimensional T1-weighted images were acquired at the UCSF using the same MRI scanner and acquisition parameters to remove effects caused by differences in MRI acquisition site and parameters. MRI data were acquired on a Siemens Tim Trio (Siemens Healthcare Inc., Erlangen, Germany) 3 Tesla MRI scanner with a 12-channel receiver head coil. Whole brain images were acquired using a volumetric magnetization-prepared rapid gradient-echo sequence using the following parameters: repetition time/echo time/inversion time = 2300/2.98/900 ms; α = 9°; sagittal orientation, 256 × 240 × 160 matrix size; and 1 mm^3^ isotropic voxel resolution. MRI data quality was centrally evaluated at the UCSF.

For external dataset, three-dimensional T1-weighted images were acquired at the Toronto, UCSD, MGH using the same MRI scanner and acquisition parameters. MRI data were acquired on a GE DISCOVERY MR 750 (GE Healthcare Inc., Milwaukee, WI, USA) 3 Tesla MRI scanner with a 8-channel receiver head coil. Whole brain images were acquired using a volumetric magnetization-prepared rapid gradient-echo sequence using the following parameters: repetition time/echo time/inversion time = 7340/3.04/400 ms; α = 11°; sagittal orientation, 196 × 256 × 256 matrix size; and 1.20 × 1.02 × 1.02 mm^3^ isotropic voxel resolution. These MRI data qualities were also centrally evaluated at the UCSF.

### MRI Processing and Volumetry

In total, 10 subregions of gray matter volume were calculated using FreeSurfer (38) version 6.0.0, including the frontal, temporal, parietal, and occipital lobes as well as the cingulate gyrus, basal ganglia, SCP, midbrain, pons, and medulla. To remove the effect of different head sizes, all regional volumes were normalized by dividing the volumes by total intracranial volume. Each regional volume was converted into a z-score relative to a control population so that the control population had a mean of 0 and a standard deviation of 1. Because *z*-scores become negative as regional brain volumes decrease with disease progression, for simplicity, we multiplied z-scores by minus one so that they increased as regional brain volumes decreased.

### Brain Atrophy Progression Modeling

We applied SuStaIn (28) to the cross-sectional regional brain volumes of internal dataset to estimate the two disease subtypes and trajectories of CBS and PSP-RS, which have distinct atrophy patterns (15, 18, 39, 40). A conceptual overview of SuStaIn modeling is shown in Figure 1. SuStaIn evaluated the optimal clustering of individuals for each disease subtype (CBS or PSP-RS) that reflected distinct patterns of brain atrophy progression based on cross-sectional regional brain volumes. Simultaneously, each progression pattern was inferred as a sequential transition of individual subregions of gray matter volume from one *z*-score to another, relative to a control population. Subtype and disease stage were determined by those with the highest likelihood of participants being assigned to each disease subtype and disease stage. The progression trajectory of each subtype was described as a linear *z*-score model (28), which was composed of a sequence of stages in which each regional brain volume followed a piecewise linear trajectory. In practice, the number of progression patterns of brain atrophy is too large to evaluate all possible progression patterns. So, we use Markov Chain Monte Carlo (MCMC) sampling, which is able to indirectly obtain inference on the posterior distribution using computer simulations (41), to provide an approximation to this uncertainty (42, 43). We used 1,000,000 MCMC samples initialized from the maximum likelihood solution.

The linear *z*-score model underlying SuStaIn is based on the event-based model (42, 43). The event-base model identifies disease progression as a series of events corresponding to a regional brain atrophy from a normal to an abnormal level in this study. For biomarker *i* = 1…*l* (i.e., brain region), the occurrence of an event *E*_*i*_ (i.e., brain atrophy) is informed by the measurements *x*_*ij*_ of biomarker *i* in subject *j*, *j* = 1…*J* (i.e., regional brain volume). The most likely ordering of the event is the sequence *S* (i.e., brain atrophy progression) that maximizes the data likelihood is described using as the whole data set *X* = {*x*_*ij*_|*i* = 1…*I, j*…*J*} as:


$$\begin{array}{l} P\left(X|S\right)=\overset{J}{\underset{j=1}{\prod }}\left[\overset{I}{\underset{k=0}{\sum }}\left(P\left(k\right)\overset{k}{\underset{i=1}{\prod }}P\left(x_{ij}|E_{i}\right)\;\overset{I}{\underset{i=k+1}{\prod }}P\left(x_{ij}|{\neg E}_{i}\right)\right)\right], \end{array}$$

where *P*(*x*_*ij*_|*E*_*i*_) and *P*(*x*_*ij*_|¬*E*_*i*_) are the likelihoods of measurement *x* given that biomarker *i* has or has not become abnormal, respectively. *P*(*k*) is the prior likelihood of being at stage *k* and, at which the events *E*_1_, …, *E*_*k*_ have occurred, and the events *E*_*k*+1_, …, *E*_*l*_ have yet to occur. The model uses a uniform prior on the stage, so that a priori individuals are equally likely to belong to any stage along the progression pattern. The likelihoods *P*(*x*_*ij*_|*E*_*i*_) and *P*(*x*_*ij*_|¬*E*_*i*_) are modeled as normal distributions from input data. The linear *z*-score model in this study reformulated the event-based model by replacing the instantaneous normal to abnormal events with events that represent the linear accumulation of a biomarker from zero to two *z*-score.

Model fitting requires simultaneously optimizing disease subtype (i.e., CBS and PSP-RS), subtype trajectory and the posterior distributions of both. The cost function depends on the sequence ordering based on the well-established methods developed for the event-based model (42–45) with convergence and optimality in simulation and in our datasets. The SuStaIn model was fitted hierarchically with the number of clusters estimated *via* model selection criteria obtained from cross-validation. The hierarchical fitting initializes the fitting of each *C*-cluster (subtype) model from the previous C-1-cluster model, that is, the clustering problem was solved sequentially from *C* = 1, 2 in this study, initializing each model using the previous model.

### Cross-Validation

Fivefold cross-validation was performed to evaluate the consistency of the disease progression patterns of each subtype by splitting the data into five folds and fitting the model to each subset (i.e., four of folds). The remaining fold was retained to test the model so that the model was fitted to each fold repeatedly, and ultimately, it was fitted to the whole internal dataset.

Model cross-validation similarity was measured using the Bhattacharyya coefficient (46) to assess the consistency of models across cross-validation folds.

The Bhattacharyya coefficient of the event position between each subregion of the gray matter volume of the two subtype progression patterns was measured by averaging across the events of subregion volume and MCMC method samples. The Bhattacharyya coefficient ranged from 0 (no similarity) to 1 (maximum similarity) for distribution similarity of event positions of subregion volumes of the subtype sequences.

### Classification of Disease Subtypes

To evaluate the classification performance of SuStaIn for CBS and PSP-RS, we calculated the classification accuracy and sensitivity of the fivefold cross-validation for CBS and PSP-RS using internal dataset. Additionally, to evaluate generalization performance of SuStaIn classification, we also calculated the classification accuracy and sensitivity of the external dataset for CBS and PSP-RS with parameters that were fitted using the internal dataset.

### Relationship Between SuStaIn Stage and Disease Severity

To evaluate the clinical utility of SuStaIn for CBS and PSP-RS, the correlation analysis was performed between the disease stage of internal dataset estimated by SuStaIn and disease severity including disease duration, total PSPRS score, PSPRS subscores, SEADL, UPDRS-III, MoCA, MMSE, CDR Box FAQ scores using Spearman rank correlation test. Additionally, to evaluate generalization performance of SuStaIn staging, we also analyzed these correlations of the external dataset that individual subject was assigned to a SuStaIn stage with parameters that were fitted using the internal dataset. Statistical significance for the correlation tests was set at 0.05.

## Results

### Demographic and Clinical Data Findings

Table 1 summarizes the demographic, clinical data and MRI findings. Groups were well-matched for age and sex distributions. The patient group in the internal dataset showed significant impairments in both motor and cognitive functions compared with the control group as shown using CDR Box data, MMSE total, MoCA total, FAQ, and SEADL scores. The PSP-RS group had significantly higher total PSPRS score and PSPRS subscores than the CBS group, with the exception of the PSPRS limb motor subscore, which showed that the CBS group was more impaired than the PSP-RS group.

In the internal dataset, overall, 84.0% (21/25) of patients with CBS had asymmetrical frontoparietal atrophy and 16.0% (4/25) showed the hummingbird sign. Four patients with CBS with the hummingbird sign also had asymmetrical frontoparietal atrophy, so the percentage of patients with CBS with only asymmetrical frontoparietal atrophy was 68.0% (17/25). In contrast, 23.1% (9/39) of patients with PSP-RS had asymmetrical frontoparietal atrophy and 64.1% (25/39) showed the hummingbird sign. Seven of 25 patients with PSP-RS with the hummingbird sign also had asymmetrical frontoparietal atrophy, so the percentage of patients with PSP-RS with only the hummingbird sign was 46.2% (18/39). In summary, the classification accuracy between CBS and PSP-RS based on classical MR signs was 54.7% (35/64) and the sensitivities for CBS and PSP-RS were 68.0% (17/25) and 46.2% (18/39), respectively.

In the external dataset, overall, 80.0% (4/5) of patients with CBS had asymmetrical frontoparietal atrophy and 20.0% (1/5) showed the hummingbird sign. One patient with CBS with the hummingbird sign also had asymmetrical frontoparietal atrophy, so the percentage of patients with CBS with only asymmetrical frontoparietal atrophy was 60.0% (3/5). In contrast, 27.3% (3/12) of patients with PSP-RS had asymmetrical frontoparietal atrophy and 63.6% (8/12) showed the hummingbird sign. Three of eight patients with PSP-RS with the hummingbird sign also had asymmetrical frontoparietal atrophy, so the percentage of patients with PSP-RS with only the hummingbird sign was 41.7% (5/12). In summary, the classification accuracy between CBS and PSP-RS based on classical MR signs was 47.1% (8/17) and the sensitivities for CBS and PSP-RS were 60.0% (3/5) and 41.7% (5/12), respectively. Thus, the external dataset had similar characters of MR findings to the internal dataset.

### Disease Subtype Progression Patterns

**Figure 2:**
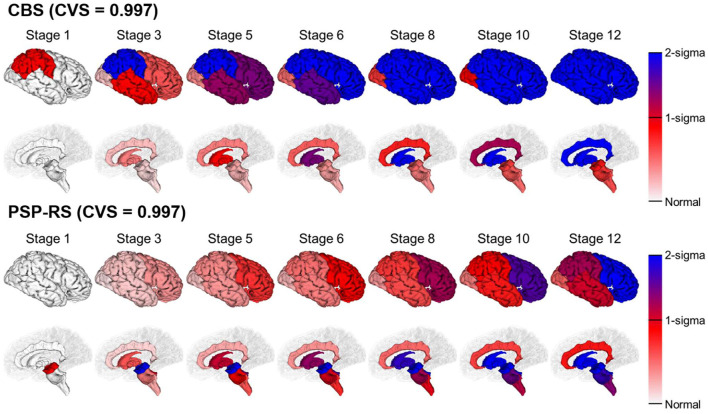
SuStaIn modeling of CBS and PSP-RS using regional brain volume data. Rows show the brain atrophy progression patterns of two subtypes estimated by SuStaIn. Each progression pattern was inferred as a sequential transition of individual subregions of gray matter volume from one z-score to another, relative to a control population. Colors at each stage and brain region indicate the severity of regional volume atrophy, where white signifies normal (z-score of 0, i.e., no atrophy), deepening to red indicates mild atrophy (z-score of 1), and deepening to blue indicates severe atrophy (z-score over 2).

Figure 2 shows the temporal progression patterns of atrophy of the CBS and PSP-RS groups obtained using SuStaIn using the internal dataset. SuStaIn identified that atrophy in patients with CBS started from the frontoparietal lobe, followed by the temporo-occipital lobe and basal ganglia, and finally reached the cingulate gyrus and brain stem. In contrast, in the PSP-RS group, atrophy started from the midbrain and SCP, followed by the pons, medulla, basal ganglia, cingulate gyrus, and frontoparietal lobe, and eventually reached the temporo-occipital lobe. These progression patterns for the two subtypes of CBS and PSP-RS were highly reproducible under cross-validation, which showed an average similarity between cross-validation folds of >99.7% for each disease subtype.

### Disease Classification

Table 2 shows the classification accuracy of SuStaIn for identifying disease subtype (CBS and PSP-RS) from cross-sectional MRI data. In the internal dataset, the classification accuracy of SuStaIn was 0.875 (56/64), and the sensitivity for CBS and PSP-RS were 0.680 (17/25) and 1.000 (39/39), respectively. In this study, the accuracy of the SuStaIn classification was superior to that of the classification based on MR findings (accuracy: 0.547 (35/64), CBS sensitivity: 0.680 (17/25), PSP-RS sensitivity: 0.462 (18/39). Moreover, four autopsy-confirmed CBD and patients with PSP-RS were correctly classified as CBS and PSP-RS, respectively. In the internal dataset, the classification accuracy of SuStaIn was 0.875 (56/64), and the sensitivity for CBS and PSP-RS were 0.600 (3/5) and 0.833 (10/12), respectively. In this study, the accuracy of the SuStaIn classification was superior to that of the classification based on MR findings (accuracy: 0.471 (8/12), CBS sensitivity: 0.600 (3/5), PSP-RS sensitivity: 0.417 (5/12). Thus, the classification accuracy of SuStaIn was superior to that of classic MR findings.

**Table 2:** Confusion matrix for the classification of CBS and PSP using SuStaIn.

	**Predicted CBS**	**Predicted PSP-RS**	**Sensitivity**
**(a) Internal dataset**			
Actual CBS	17	8	0.680 (17/25)
Actual PSP-RS	0	39	1.000 (39/39)
Precision	1.000 (17/17)	0.830 (39/48)	**Accuracy 0.875** (56/64)
	**Predicted CBS**	**Predicted PSP-RS**	**Sensitivity**
**(b) External dataset**			
Actual CBS	3	2	0.600 (3/5)
Actual PSP-RS	2	10	0.833 (10/12)
Precision	0.600 (3/5)	0.833 (10/12)	**Accuracy 0.765** (13/17)

*HC, healthy control; CBS, corticobasal syndrome; PSP-RS, progressive supranuclear palsy Richardson's syndrome*.

**Figure 3:**
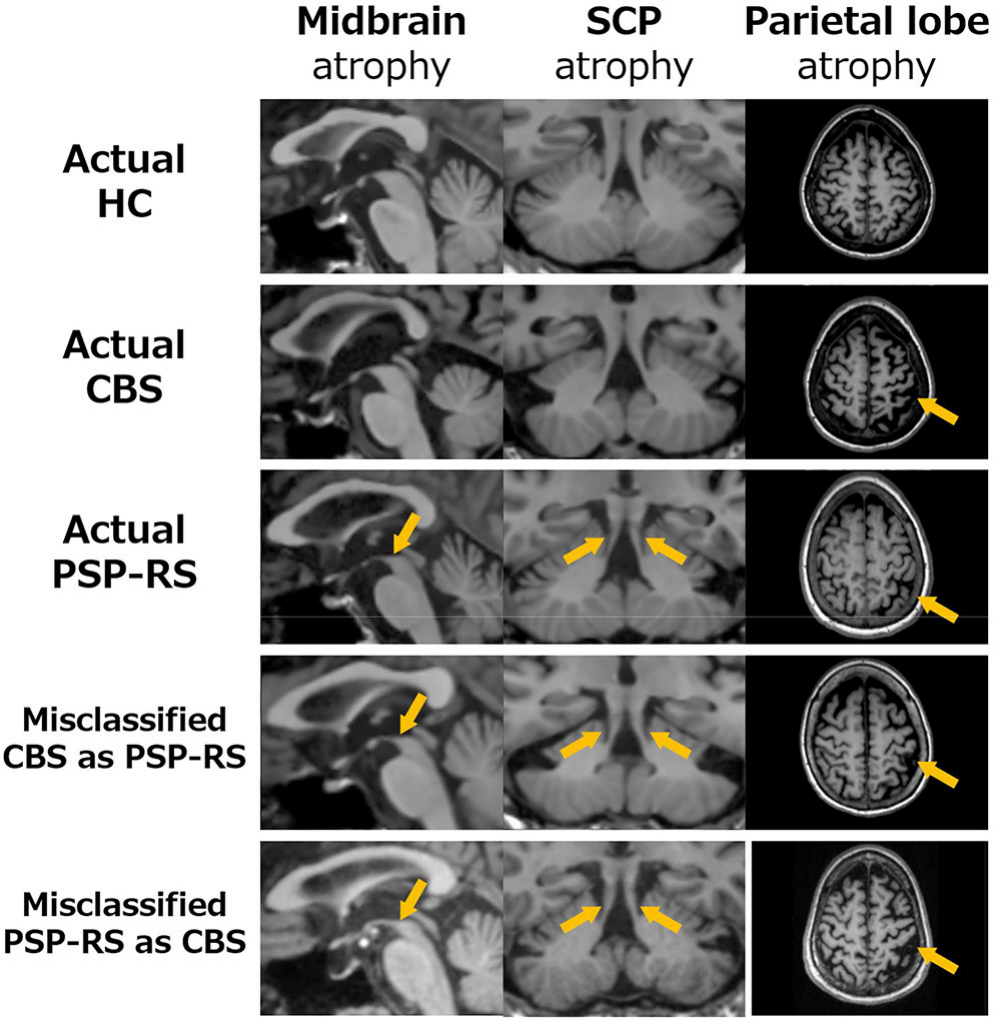
Comparison the brain atrophy in midbrain (left), SCP (middle) and parietal lobe (right) among classified patients into CBS and PSP-RS by the SuStaIn. In patients with CBS misclassified as PSP-RS (4th raw), the atrophies of the midbrain (1st column) and SCP (2nd column) were similar to PSP-RS (3rd raw), in which atrophy of the midbrain and SCP is severe (orange arrows). Furthermore, atrophy of the parietal lobe (3rd column), which is typically severe in CBS, in patients with CBS misclassified as PSP-RS was much weaker than that in correctly classified CBS and very close to that in correctly classified PSP-RS (orange arrows) patients. Similarly, patients with PSP-RS misclassified as CBS (5th raw) resembled the atrophy characters of patients with CBS (2nd raw) and the parietal lobe in patients with PSP-RS misclassified as CBS was much stronger than that in correctly classified PSP-RS.

Although SuStaIn classified CBS and PSP-RS as individual disease subtypes with high accuracy, it misclassified CBS as PSP-RS and PSP-RS as CBS, in some patients. To investigate the reason for SuStaIn misclassification, the remarkable regional parietal lobe atrophy in patients with CBS and midbrain and SCP atrophy in patients with PSP-RS were compared between the patient groups classified by SuStaIn (i.e., correctly classified CBS and PSP-RS vs. misclassified CBS as PSP-RS) based on the *z*-score, relative to HCs with a mean of 0 and a standard deviation of 1. The PSPRS total scores were also compared among these patient populations. Figure 3 and Table 3 show the differences in regional brain atrophy among the HC, correctly classified CBS and PSP-RS, misclassified CBS as PSP-RS groups and misclassified PSP-RS as CBS groups. The characteristics of regional brain atrophy and the PSPRS total scores in mis-classified patients with CBS were close to those of the correctly classified patients with PSP-RS, while the characteristics of regional brain atrophy and the PSPRS total scores in mis-classified patients with PSP-RS also were close to those of the correctly classified patients with CBS. Notably, patients with CBS classified as PSP-RS showed the remarkable MR finding of the hummingbird sign which is characteristic of PSP-RS. Similarly, patients with PSP-RS classified as CBS showed the remarkable MR finding of the asymmetrical frontoparietal atrophy which is characteristic of CBS.

**Table 3:** Comparison of brain atrophy in the parietal lobe, midbrain, and SCP and PSPRS total score among patients classified as CBS and PSP-RS by SuStaIn.

	**Actual**	**Misclassified**	**Actual**	**Misclassified**
	**CBS**	**CBS**	**PSP-RS**	**PSP-RS**
**(a) Internal dataset**
*n*	17	8	39	*none*
Parietal lobe (*z*-score)	2.72 ± 1.18	0.74 ± 1.21	0.86 ± 1.11	*none*
Midbrain (*z*-score)	0.18 ± 1.02	1.47 ± 1.06	2.90 ± 0.84	*none*
SCP (*z*-score)	−0.08 ± 1.31	1.18 ± 1.85	2.16 ± 1.24	*none*
PSPRS Total	24.6 ± 10.4	27.8 ± 6.9	36.0 ± 16.0	*none*
**(b) External dataset**
*n*	3	2	10	2
Parietal lobe (*z*-score)	2.96 ± 1.01	−1.55 ± 0.23	0.38 ± 0.86	2.23 ± 0.46
Midbrain (*z*-score)	−0.73 ± 1.05	0.91 ± 2.24	2.36 ± 0.66	3.03 ± 0.43
SCP (*z*-score)	−0.07 ± 1.27	1.27 ± 1.65	1.72 ± 1.86	−0.26 ± 1.49
PSPRS Total	20.3 ± 4.50	31.0 ± 3.00	31.4 ± 15.9	63.0 ± 7.00

*HC, healthy control; CBS, corticobasal syndrome; PSP-RS, progressive supranuclear palsy Richardson's syndrome, PSPRS, PSP Rating Scale. Each regional volume was converted to a z-score relative to a control population so that the control population had a mean of 0 and a standard deviation of 1. Because z-scores become negative as regional brain volumes decrease with disease progression, for simplicity, we multiplied the z-scores by −1 so that they increased as regional brain volumes decreased*.

### Relationship Between SuStaIn Stage and Severity

Table 4 shows the correlation (*r*_*s*_) between the disease stage as estimated by SuStaIn and disease severity in CBS and PSP-RS. The results of the internal data set for CBS and PSP-RS showed a significant correlation between SuStaIn stage and disease severity, except for PSPRS Ocular Motor (*P* = 0.939). However, the correlation with disease duration only tended toward significance (*P* < 0.058). The significant relationship between the SuStaIn stage and disease severity in CBS was indicated in the FAQ, CDR Box, and SEADL score, whereas that in PSP-RS was indicated in all scores except for the FAQ and CDR Box. Although there were no significant correlations between cognitive function and MoCA or MMSE scores in CBS, the relationship tended toward significance (MoCA, *P* = 0.071; MMSE, *P* = 0.066). The total PSPRS score and subscore were significantly correlated with SuStaIn stage only in PSP-RS. The increment in disease duration, PSPRS, UPDRS-III, FAQ, and CDR Box scores as well as the decrement in SEADL, MoCA, and MMSE scores reflect high disease severity. This tendency was similar to the results of the external data. Thus, SuStaIn stage in CBS and PSP-RS was significantly related to disease severity and correctly reflected the same.

**Table 4:** Relationship between disease severity and SuStaIn stage.

**Information**	**CBS and PSP-RS**		**CBS**		**PSP-RS**	
	***r_***s***_***	***P-*value**	***r_***s***_***	***P*-value**	***r_***s***_***	***P*-value**
**(a) Internal dataset**
Disease duration	0.25	0.058	0.02	0.934	0.41	<0.05
PSPRS total	0.50	<0.001	0.11	0.658	0.55	<0.005
PSPRS bullbar	0.37	<0.01	−0.04	0.864	0.41	<0.05
PSPRS ocular–motor	0.44	<0.005	−0.17	0.471	0.41	<0.05
PSPRS limb–motor	−0.01	0.939	−0.25	0.285	0.50	<0.005
PSPRS gait/midline	0.51	<0.001	0.41	0.075	0.49	<0.005
UPDRS-III total	0.31	<0.05	0.36	0.089	0.41	<0.05
FAQ total	0.41	<0.005	0.45	<0.05	0.29	0.111
CDR box	0.39	<0.005	0.57	<0.005	0.29	0.083
SEADL	−0.61	<0.001	−0.56	<0.05	−0.67	<0.001
MoCA total	−0.33	<0.05	−0.41	0.071	−0.39	<0.05
MMSE total	−0.31	<0.05	−0.41	0.066	−0.33	<0.05
**(b) External dataset**
Disease duration	*none*	*none*	*none*	*none*	*none*	*none*
PSPRS total	0.63	<0.01	0.74	0.155	0.70	<0.05
PSPRS bullbar	0.46	0.065	0.49	0.406	0.47	0.120
PSPRS ocular–motor	0.36	0.169	0.87	0.058	0.72	<0.05
PSPRS limb–motor	0.72	<0.005	−0.16	0.794	0.86	<0.001
PSPRS gait/midline	0.42	0.104	0.38	0.530	0.62	<0.05
UPDRS-III total	0.95	<0.001	0.97	<0.005	0.95	<0.001
FAQ total	0.60	<0.05	0.45	0.453	0.65	<0.05
CDR box	0.54	<0.05	0.94	0.057	0.52	0.082
SEADL	−0.59	<0.05	−0.97	<0.005	−0.50	0.116
MoCA total	−0.64	<0.05	−0.94	0.057	−0.79	<0.01
MMSE total	−0.71	<0.005	−0.87	0.333	−0.79	<0.005

*HC, healthy control; CBS, corticobasal syndrome; PSP-RS, progressive supranuclear palsy Richardson's syndrome; PSPRS, PSP Rating Scale; Four motor subscores from total PSPRS, PSPRS Bulbar, PSPRS Ocular Motor, PSPRS Limb Motor, and PSPRS Gait/Midline; UPDRS-III, Part-III (motor exams) of the Unified Parkinson's Disease Rating Scale; CDR Box, Clinical Dementia Rating Sum of Boxes; MMSE, Mini-Mental State Examination; MoCA, Montreal Cognitive Assessment; FAQ, Functional Activities Questionnaire; SEADL, Schwab and England Activities of Daily Living*.

## Discussion

In this study, we used SuStaIn, a recently developed unsupervised machine learning technique for data-driven disease phenotype discovery, to clarify the differences in the temporal progression patterns of brain atrophy between CBS and PSP-RS for the first time. SuStaIn successfully revealed distinct brain atrophy progression patterns that corresponded to CBS and PSP-RS, with a high average similarity across cross-validation folds for each disease subtype. The progression model of CBS showed that brain atrophy started from the cerebral neocortex, especially the frontoparietal lobe, followed by the cingulate gyrus and basal ganglia, and finally reached the brain stem. In contrast, PSP-RS progression showed atrophy starting from the brainstem, specifically the midbrain and SCP, followed by the basal ganglia and cingulate gyrus, and eventually reaching the cerebral neocortex. Furthermore, SuStaIn classified CBS and PSP-RS as individual disease subtypes with high accuracy. Additionally, the disease stage of individual patients with CBS and PSP-RS had significant relation with the disease severity and correctly reflect it.

Our estimated atrophy progression of CBS and PSP-RS using SuStaIn is largely consistent with previous histopathological studies. In a postmortem study investigating the distribution and severity of tau pathology in preclinical and end-stage CBD, changes in neuronal lesions were largest in the frontal and parietal cortices, moderate in the basal ganglia, and mild in the brainstem (47). Another postmortem study that investigated the distribution of tau pathology in PSP-RS showed sequential distribution patterns that suggested the accumulation of different cellular tau pathologies in PSP-RS (48). The sequence of PSP-RS related neuronal tau pathology started from the substantia nigra, followed by the midbrain tegmentum, medulla oblongata, pons base, and frontal lobe, and eventually reached the parietal, temporal, and occipital lobes (48). This suggests that temporal atrophy progression patterns identified using SuStaIn mirror the progression of tau pathology in CBD-CBS and PSP-RS.

Our finding that temporal atrophy progression in PSP-RS starts from the SCP (49) and that of CBS starts from the frontoparietal cortex (50) is consistent with histopathological reports on cerebral tau accumulation. The SCP comprises the dentate nucleus in the cerebellum that ascends to the ventrolateral thalamus through the SCP. Degeneration and activated microglia along this tract have been shown to be the specific characteristics of PSP-RS pathology (51, 52). In our atrophy progression model of PSP-RS, we found other prominent regions of atrophy in the early stages, such as the midbrain, followed by basal ganglia. These regions are consistent with neuropathological distributions of tau-positive astrocytic inclusions observed in patients with PSP-RS (53). Unlike the histopathological features of PSP-RS, which are characterized by neuronal loss, gliosis, and abundant neurofibrillary tangles in the midbrain and SCP (2), the extensive accumulation of tau-immunoreactive inclusions and astrocytic plaques in the gray and white matter is a distinct characteristic of CBD pathology (54).

In line with the postmortem studies, previous cross-sectional brain volumetric studies using structural MRI have revealed midbrain and SCP atrophy as the primary features of PSP-RS (15, 18, 39, 40, 55, 56). In contrast, greater atrophy is observed in the frontoparietal cortex in CBS (16, 19). A meta-analysis of voxel-based morphometry studies showed more prominent atrophy in the superior parietal lobe in CBS compared with PSP-RS, although there was significant overlap of atrophy between PSP-RS and CBS (57). In a pathology-proven sample, atrophy of the midbrain and SCP was strongly associated with PSP-RS, whereas frontoparietal degeneration without significant brainstem atrophy was more implicative of CBD-CBS (18). Atrophy progression estimated using SuStaIn reflected regional changes in brain areas that are known to be most severely affected by CBD and PSP-RS pathology, which included the frontoparietal cortex and midbrain/SCP, respectively. These regions showed the earliest longitudinal changes in patients with CBS and PSP-RS.

The temporal brain atrophy progression patterns of patients with CBS and those with PSP-RS estimated by SuStaIn were considerably consistent with those of previous longitudinal studies. A 1-year longitudinal study, which used voxel-based morphometry of structural MRI data, (24) reported that the regional volumes of patients with CBS compared with HCs at baseline showed atrophy in the precentral gyrus, supplementary motor cortex, and postcentral gyrus. Regional brain atrophy extended to the putamen and pallidum of the basal ganglia over 6 months. Finally, atrophy reached the midbrain and pons at 12 months. For patients with PSP-RS compared with HCs at baseline, the most remarkable regional atrophy occurred in the midbrain, pontine tegmentum, and SCP. Over 1-year, cortical atrophy extended over the frontoparietal and occipital lobes, accompanied by atrophy of the basal ganglia, which included the putamen and pallidum. Additionally, Zhang et al. (56) investigated the progression of microstructural degeneration in CBS and PSP-RS using diffusion tensor imaging, which is sensitive to more subtle brain pathology. Results showed that the most prominent changes in PSP-RS were in the SCP, followed by the basal ganglia, whereas those in CBS were in the basal ganglia as well as widespread white matter regions, which included the frontal, parietal, occipital, and temporal lobes.

We compared the classification accuracies of SuStaIn and classic MR signs evaluated by an expert neuroradiologist in this study with those of a previous study (11–14, 16, 27, 58, 59) in the differentiation of CBS and PSP-RS. In the present study, the classification accuracy of internal dataset between CBS and PSP-RS based on the expert neuroradiologist's diagnosis from MR findings was 0.547 (35/64) and the sensitivities for CBS and PSP-RS were 0.680 (17/25) and 0.462 (18/39), respectively, and additionally, the classification accuracy of internal dataset was similar to that of external dataset (accuracy, 47.1% (8/17); sensitivity for CBS, 60.0% (3/5); sensitivity for PSP-RS, 41.7% (5/12). It was reported that the sensitivity and specificity according to classical morphological markers of PSP-RS on MRI were approximately 0.510 and 0.995 based on the “hummingbird” sign and a~0.370 and 0.970 based on the “morning glory” sign, respectively (11–13). Thus, these two signs had high specificity but low sensitivity to distinguish PSP-RS and CBS (14). Overall, the classification performance of SuStaIn was superior to that of classic MR signs in terms of accuracy, sensitivity, and specificity. There were some reasons for this finding. A previous study using MRI volumetry reported that midbrain atrophy was associated with the clinical presentation of PSP-RS but not with the pathological diagnosis of PSP in the absence of clinical PSP-RS (23, 60). Similarly, asymmetric atrophy of the frontal lobe, including the premotor cortex, which is common in CBS, was not associated with background neuropathologies. In fact, previous studies reported midbrain atrophy in patients with pathologically proven CBD and asymmetric frontal atrophy in patients with pathologically proven PSP (23, 61, 62). Thus, the classification performance of classic MR signs could be lower than that of SuStaIn, because there is some heterogeneity in typical brain atrophy based on MR findings in CBS and PSP-RS.

In a previous study using a classifier, Gröschel et al. used the mathematical model for brain MR volumetry, which included the midbrain, parietal white matter, temporal gray matter, brainstem, frontal white matter, and pons in patients with PSP-RS (*n* = 33) and CBD-CBS (*n* = 18) (16). The model correctly predicted PSP-RS and CBD-CBS with 76 and 83% sensitivity, respectively. Correia et al. used a SVM method on gray matter volume data to classify 19 patients with PSP-RS and 19 patients with CBS (27). Using a leave-two-out cross-validation approach, the mean classification accuracy of the SVM was found to be 62.2%. Also using a leave-two-out cross-validation approach, Correia et al. also observed 79.8% mean accuracy using diffusion tensor image data, which included measures of fractional anisotropy and mean diffusivity. There are also studies that have used other modalities. One study used a semiquantitative 123I-N-ω-fluoropropyl-2β-carbomethoxy-3β-(4-iodophenyl) nortropane (123I-FP-CIT) single-photon emission computed tomography striatal evaluation combined with SVM (58) that differentiated 41 patients with PSP-RS and 28 patients with CBS with 73.9% accuracy (PSP-RS sensitivity and specificity of 82.6 and 72.7%, respectively). Another study used an assay of cerebrospinal fluid (CSF) tau (59, 63), which reported 84.2% sensitivity and 66.7% specificity for separating 21 patients with PSP-RS and 12 patients with CBS using the p-tau/t-tau ratio. Urakami et al. obtained 81.5% sensitivity and 80% specificity for classifying CBS and PSP-RS based on tau protein levels in the CSF using sandwich enzyme linked immunosorbent assay. The classification accuracy of our study using SuStaIn based on only structural MRI was higher than previous studies and suggested that SuStaIn modeling for disease-stage heterogeneity allows better stratification of CBS and PSP-RS compared with models that only predict disease subtypes (28). Furthermore, in this study, the classification accuracy of external dataset was similar to that of internal dataset and it suggested that SuStaIn had the generalization performance of classification for CBS and PSP-RS. We will look at including other modalities in future work to uncover disease progression of CBS and PSP-RS and stratify these diseases with higher accuracy and reliability.

Although SuStaIn classified CBS and PSP-RS as individual disease subtypes with high accuracy, it misclassified CBS as PSP-RS in some patients. Notably, half of these patients with CBS misclassified as PSP-RS showed the hummingbird sign on MRI. As shown in Figure 3 and Table 3, in patients with CBS misclassified as PSP-RS, atrophy of the midbrain and SCP was similar to that in correctly classified PSP-RS cases, where atrophy of the midbrain and SCP is severe (15, 18, 39, 40), and the PSP-RSRS total score approached that of correctly classified patients with PSP-RS. Furthermore, atrophy of the parietal lobe in patients with CBS misclassified as PSP-RS (15, 16) was much weaker than that in correctly classified patients with CBS and very close to that in correctly classified patients with PSP-RS. Thus, the above results suggest that the eight patients with CBS misclassified as PSP-RS, by SuStaIn, had atrophy characteristics strongly associated with PSP-RS, which may have resulted in the misclassification. Importantly, CBD pathology is observed not only in CBS (~50%) but also in PSP-RS, Alzheimer's disease (AD), frontal temporal lobar degeneration (FTLD), and Creutzfeldt–Jakob disease (64). Although CBD pathology is determined by the Armstrong criteria and has four phenotypes in including CBS, frontal behavioral-spatial syndrome (FBS), nonfluent/agrammatic variant of primary progressive aphasia (naPPA), and PSP syndrome (PSPS) (7), a postmortem pathology study using the antemortem Armstrong criteria (65)—reported that the accuracy of diagnosis was 68% (13 of 19 CBD patients) and that these patients with CBS met the criteria for probable CBD; however, all patients (14/14) with CBS without CBD pathology, such as AD and FTLD pathologies, met the criteria for probable or possible CBD. These results imply that the Armstrong criteria lack the necessary specificity for accurate antemortem clinical diagnosis of CBD.

As shown in Table 4, the disease stage of individual patients with CBS and PSP-RS was significantly related to disease severity. The increment in disease duration, PSPRS, UPDRS-III, FAQ, and CDR Box scores as well as the decrement in SEADL, MoCA, and MMSE scores reflect high disease severity. The significant relationship between SuStaIn stage and disease severity in CBS was indicated in the FAQ, CDR Box, and SEADL scores, whereas that in PSP-RS was indicated in all scores except for the FAQ and CDR Box. In the internal data set, the correlations between cognitive function and MoCA and MMSE scores in CBS only tended toward significance (MoCA, *P* = 0.071; MMSE, *P* = 0.066). Similarly, the correlation between motor function and PSPRS Gait/Midline and UPDRS-III scores in PSP-RS only tended toward significance (PSPRS Gait/Midline, *P* = 0.075; UPDRS-III, *P* = 0.089). In this study, the sample size of the CBS group (*n* = 25) was smaller than that of the PSP-RS group (*n* = 39). Therefore, this insufficient sample size might have affected the significance of the correlation test. Moreover, taking into consideration that the correlation coefficients (*r*_*s*_) of the clinical scores related to cognitive and motor function were quite similar between CBS (MoCA, *r*_*s*_ = −0.41; MMSE, *r*_*s*_ = −0.41; PSPRS Gait/Midline, *r*_*s*_ = 0.41; UPDRS-III, *r*_*s*_ = 0.36) and PSP-RS (MoCA, *r*_*s*_ = −0.39; MMSE, *r*_*s*_ = −0.33; PSPRS Gait/Midline, *r*_*s*_ = 0.49; UPDRS-III, *r*_*s*_ = 0.41) and because the overlap in CBS and PSP-RS between clinical symptoms including cognitive and motor function is well-known, the SuStaIn stage could reflect exactly the disease severity of cognitive function. The total PSPPR score and subscore were significantly correlated with SuStaIn stage in only PSP-RS. A previous study on clinical correlations between annual change in PSPRS score and loss in regional brain volume demonstrated a tendency toward a significant correlation in the PSP-RS group but not in the CBS group (24, 66). The results of this study provide support for the findings of previous research. Thus, SuStaIn stage in CBS and PSP-RS was significantly associated with disease severity and correctly reflected it without the label of disease stage, such as disease duration. Moreover, in this study, the above tendency of the internal data set was similar to the result of the external data set, and it suggests that SuStaIn generally reflects the disease severity in patients with CBS and PSP-RS. Furthermore, SuStaIn could be used to stratify patients with CBS or PSP-RS and predict patient prognoses using only brain structural characters based on cross-sectional MRI.

The present study is limited by the fact that the diagnoses of our patients were based on clinical symptoms without autopsy confirmation. Although clinical criteria accurately stratified PSP-RS pathology, they have been shown to be insufficiently specific for CBD pathology and there is a heterogeneous condition consisted of not only CBD but also various neurodegenerative disorders such as PSP, AD, TDP-43 proteinopathy, Pick disease, and dementia with Lewy bodies (7). So, there was possible to inclusion of a mixture of various neuropathologies in clinically-diagnosed CBS. Therefore, the inclusion of patients with CBS who may have had various neuropahologies except for CBD pathology results may have biased our findings of the CBS group. We did not confirm whether our patients with CBS had these various neuropahologies based on negative results from amyloid, dopamine transporter or 18F-fluorodeoxyglucose imaging or CSF analysis and did not exclude patients with CBS who had PSP, AD, TDP-43 proteinopathy, Pick disease, and dementia with Lewy bodies pathology. Therefore, additional studies that exclude positive cases of these imaging will be necessary to avoid the effect of the heterogeneity of CBS neuropathologies. Lastly, further studies of autopsy-confirmed CBD and PSP-RS cases are needed to definitively determine differences in brain atrophy progression between these conditions.

While pioneering studies investigated longitudinal brain atrophy using longitudinal brain structural MRI at a few time points based on the assumption that volumetric changes are linear (24–26), this study performed SuStaIn, a recent unsupervised machine learning innovation that integrates clustering and disease progression modeling based on widely available cross-sectional data, for cross-sectional brain structural MRI to identify temporal atrophy progressions with over a few stages as Figure 1 shows and thus had more information on the longitudinal atrophy of CBS and PSP-RS. Moreover, we estimated the temporal atrophy progression patterns of each disease subtype based on widely available cross-sectional data without disease labels, and the results were largely consistent with previous studies. Therefore, SuStaIn has the potential to be a useful tool for longitudinal studies to uncover data-driven disease phenotypes with distinct temporal progression patterns. Our findings advance our understanding of the temporal atrophy progression patterns of PSP-RS and CBS and are the first to apply such a data-driven modeling approach to study CBS and PSP-RS.

## Conclusion

The present study is the first report that applied SuStaIn to clarify brain atrophy progression of CBS and PSP-RS. SuStaIn identified two disease subtypes and trajectories of CBS and PSP-RS with distinct atrophy patterns using cross-sectional regional brain volume data. Furthermore, the temporal progression patterns of brain atrophy in CBS and PSP-RS estimated by SuStaIn were largely consistent with previous reports, and the classification accuracy of CBS and PSP-RS was higher than those of previous studies. Although the disease mechanisms remain poorly understood, SuStaIn may be a promising tool to achieve accurate patient stratification and prognostication to develop effective treatment methods for PSP-RS and CBS.

## Data Availability Statement

The original contributions presented in the study are included in the article/supplementary material, further inquiries can be directed to the corresponding author.

## Ethics Statement

Ethical review and approval was not required for the study on human participants in accordance with the local legislation and institutional requirements. The patients/participants provided their written informed consent to participate in this study.

## Author Contributions

YS, KK, and KS conceived the presented idea. YS developed the theory and performed the computations. PW and DA verified the analytical methods. KK encouraged YS to investigate brain atrophy progression in PSP and CBS and supervised the findings of this work. All authors discussed the results and contributed to the final manuscript.

## Funding

This study was partially supported by the Juntendo Research Branding Project, JSPS KAKENHI (grant nos. JP21K07690, JP18H02772, and 19K17244), a Grant-in-Aid for Special Research in Subsidies for ordinary expenses of private schools from The Promotion and Mutual Aid Corporation for Private Schools of Japan, and the Brain/MINDS Beyond program (grant no. JP21dm0307101) of the Japan Agency for MedicalJ Research and Development. PW was supported by an MRC Skills Development Fellowship (MR/T027770/1). JEPSRC grant EP/M020533/1, JPND E-DADS MRC grant MR/T046422/1, and Wellcome Trust Investigator in Science award UNS113739 support DCA's work on this topic.

## Conflict of Interest

The authors declare that the research was conducted in the absence of any commercial or financial relationships that could be construed as a potential conflict of interest.

## Publisher's Note

All claims expressed in this article are solely those of the authors and do not necessarily represent those of their affiliated organizations, or those of the publisher, the editors and the reviewers. Any product that may be evaluated in this article, or claim that may be made by its manufacturer, is not guaranteed or endorsed by the publisher.
